# RNA interference-based resistance in transgenic tomato plants against *Tomato yellow leaf curl virus*-Oman (TYLCV-OM) and its associated betasatellite

**DOI:** 10.1186/s12985-015-0263-y

**Published:** 2015-03-04

**Authors:** Um e Ammara, Shahid Mansoor, Muhammad Saeed, Imran Amin, Rob W Briddon, Abdullah Mohammed Al-Sadi

**Affiliations:** Department of Crop Sciences, College of Agriculture and Marine Sciences, Sultan Qaboos University, P.O. Box-34, 123 Al-Khod, Oman; Agricultural Biotechnology Division, National Institute for Biotechnology and Genetic Engineering (NIBGE), P O Box 577, Jhang Road, Faisalabad, Pakistan

**Keywords:** Begomovirus, Betasatellite, Resistance, Transformation, RNAi

## Abstract

**Background:**

*Tomato yellow leaf curl virus* (TYLCV), a monopartite begomovirus (family *Geminiviridae*) is responsible for heavy yield losses for tomato production around the globe. In Oman at least five distinct begomoviruses cause disease in tomato, including TYLCV. Unusually, TYLCV infections in Oman are sometimes associated with a betasatellite (Tomato leaf curl betasatellite [ToLCB]; a symptom modulating satellite). RNA interference (RNAi) can be used to develop resistance against begomoviruses at either the transcriptional or post-transcriptional levels.

**Results:**

A hairpin RNAi (hpRNAi) construct to express double-stranded RNA homologous to sequences of the intergenic region, coat protein gene, V2 gene and replication-associated gene of *Tomato yellow leaf curl virus*-Oman (TYLCV-OM) was produced. Initially, transient expression of the hpRNAi construct at the site of virus inoculation was shown to reduce the number of plants developing symptoms when inoculated with either TYLCV-OM or TYLCV-OM with ToLCB-OM to *Nicotiana benthamiana* or tomato. *Solanum lycopersicum* L. cv. Pusa Ruby was transformed with the hpRNAi construct and nine confirmed transgenic lines were obtained and challenged with TYLCV-OM and ToLCB-OM by *Agrobacterium*-mediated inoculation. For all but one line, for which all plants remained symptomless, inoculation with TYLCV-OM led to a proportion (≤25%) of tomato plants developing symptoms of infection. For inoculation with TYLCV-OM and ToLCB-OM all lines showed a proportion of plants (≤45%) symptomatic. However, for all infected transgenic plants the symptoms were milder and virus titre in plants was lower than in infected non-transgenic tomato plants.

**Conclusions:**

These results show that RNAi can be used to develop resistance against geminiviruses in tomato. The resistance in this case is not immunity but does reduce the severity of infections and virus titer. Also, the betasatellite may compromise resistance, increasing the proportion of plants which ultimately show symptoms.

**Electronic supplementary material:**

The online version of this article (doi:10.1186/s12985-015-0263-y) contains supplementary material, which is available to authorized users.

## Background

Tomato (*Solanum lycopersicum* L.) is the sixth largest horticulture crop grown in tropical and subtropical regions [1]. Tomato yellow leaf curl disease (TYLCD) has become the key limiting factor for the production of tomato in many areas [2]. Since the first report of TYLCD by Cohen [3] in Israel, the virus causing the disease has spread and now causes heavy yield losses worldwide [4].

*Tomato yellow leaf curl virus* (TYLCV) is a monopartite begomovirus (genus *Begomovirus*, family *Geminiviridae*), with a genome of 2.6-2.8 kb, that is transmitted by the whitefly *Bemisia tabaci*. Geminiviruses replicate via a double-stranded (ds) DNA intermediate and transcription occurs in a bidirectional manner from a non-coding intergenic region (IR). The genomes of monopartite begomoviruses from the OW encode six proteins. The genes in the virion-sense encode the coat protein (CP; involved in virus movement in and between plants) and pre-coat protein (a virulence determinant, suppressor of gene silencing and involved in virus movement in plants) [5-9]. The complementary-sense strand encodes the replication-associated protein (Rep; the only virus-encoded protein required for viral DNA replication that is a rolling-circle replication [RCR] initiator protein), the transcriptional activator protein (TrAP; involved in up-regulating late gene expression and may be a suppressor of gene silencing), the replication enhancer protein (REn), and the C4 protein (a pathogenicity determinant which may act as a suppressor of gene silencing) [7,10-12]. The IR region (~300 nt) contains the origin of virion-strand DNA replication consisting of a predicted stem-loop structure containing the conserved (among all geminiviruses) nonanucleotide sequence TAATATTAC (which is nicked by the Rep to initiate RCR) and high affinity Rep recognition sequences known as iterons [13,14]. Begomoviruses in the OW are often associated with a class of DNA satellite molecules referred to as betasatellite [15]. Betasatellites are true satellites that require a helper virus for their replication and movement in plants and transmission between plants [16,17].

TYLCV-like symptoms in Oman were first observed in late 80’s by the Ministry of Agriculture and Fisheries (MAF), although the causative agent was not identified as TYLCV until 2007 [18]. The virus identified in Oman showed the highest levels of nucleotide sequence identity (91%) to TYLCV and is a distinct strain of the species; the Oman strain of TYLCV (TYLCV-OM). Additionally, a betasatellite was identified in association with TYLCV-OM having 88.5% nucleotide sequence identity with Tomato leaf curl betasatellite (ToLCB) isolated from Pakistan [18].

In Oman, farmers use three major management practices to control TYLCV; insecticides, a physical barrier (AGRYL™ cover) and partially resistant tomato cultivars. Despite these control measures the incidence of TYLCV may at times reach 100% on some farms in Oman [18]. Conventional breeding is considered the simplest and most reliable strategy for obtaining resistance [19]. However, conventional breeding is a time consuming procedure and sometimes may lead to undesirable characteristics due to linkage drag [20]. However, conventional breeding has not been entirely effective in controlling crop losses due to begomoviruses or the whitefly vector. Genetic engineering has the potential to address this issue and offers an alternative route to virus resistance in plants. RNA interference (RNAi) provides a possible solution to address the control of diseases caused by begomoviruses [21,22].

RNAi (also known as gene silencing) is an evolutionarily conserved mechanism for down-regulating gene expression in a sequence-specific manner that is triggered by double-stranded (ds)RNA. Begomoviruses are targeted by gene silencing both at the transcriptional level (transcriptional gene silencing [TGS]), that results in viral DNA methylation, and the post-transcriptional level (post-transcriptional gene silencing [PTGS]), which results in degradation of viral transcripts. Both TGS and PTGS involve a dsRNA trigger that is cleaved into short interfering (si)RNAs by an RNase referred to as Dicer. The siRNAs then provide the sequence specificity for silencing. For PTGS siRNAs are incorporated into an enzyme complex, the RNA-induced silencing complex (RISC), which degrades mRNAs homologous to the incorporated siRNA [23-26]. The introduction into plants of a sequence homologous to the virus, in the form of an inverted repeat hairpin (hp) construct, is an efficient method to provide virus resistance in plants by inducing gene silencing [27]. The success of RNAi based resistance relies on a silencing signal which is not only limited to individual cell, but can spread from the initially infected cells to more distant tissues [28,29].

Although geminiviruses have no dsRNA stage in their replication cycle they do induce the production of virus-specific siRNA and have been shown to trigger PTGS in infected plants [30,31]. An increased accumulation of cassava-infecting geminivirus-derived siRNAs in infected cassava is associated with a corresponding decrease in disease symptom severity [30], providing a clue for RNAi as an adaptive defense against geminiviral infection in plants. Unlike the RNA viruses, which are affected by only PTGS, geminiviruses are affected by both PTGS and TGS. TGS is triggered when siRNA homologous to the promoter regions are produced, leading to inhibition of transcription due to methylation of promoter sequences [32]. TGS was shown to be effective against the begomovirus *Mungbean yellow mosaic virus* (MYMV) in a transient assay in which it was shown that MYMV-infected black gram (*Vignamungo*) plants showed complete recovery from infection after inoculation of an RNAi construct targeting viral promoter sequences in the IR [33]. Recently RNAi-based resistance has been successfully applied in beans against *Bean golden mosaic virus* (BGMV) in Brazil. Transgenic bean lines were tested in the field and showed immunity to BGMV infection. These transgenic beans are now available to farmers for cultivation; the first commercially available RNAi-based resistance in a crop against a geminivirus [34,35].

The study described here has investigated the hpRNAi strategy as a means to control the TYLCV complex in Oman by targeting four regions of the TYLCV-OM genome. The hpRNAi construct was transformed in *S. lycopersicum L.* plants by *Agrobacterium*-mediated transformation. Resistance was evaluated in transgenic tomato plants by challenging with TYLCV-OM and ToLCB. The potential of multi-targeted hpRNAi strategy for delivering resistance to begomoviruses is discussed.

## Results

### Evaluation of the resistance imparted by the hpRNAi construct by transient assay

A multi-target hpRNAi construct containing sequences of the Rep gene, IR region, V2 and overlapping CP genes of TYLCV-OM was produced. This consisted of 112 bp of IR (coordinates19-128), 161 bp of V2 (coordinates 129-288), 127 bp of the overlapping CP and 175 bp (coordinates 1760-1934) of the Rep. The 575 bp fragment was inserted in the expression vector in sense and antisense orientation separated by an intron to form a hairpin-loop structure. The whole RNAi cassette was expressed from a double *Cauliflower mosaic virus* (CaMV) 35S promoter.

**Figure 1:**
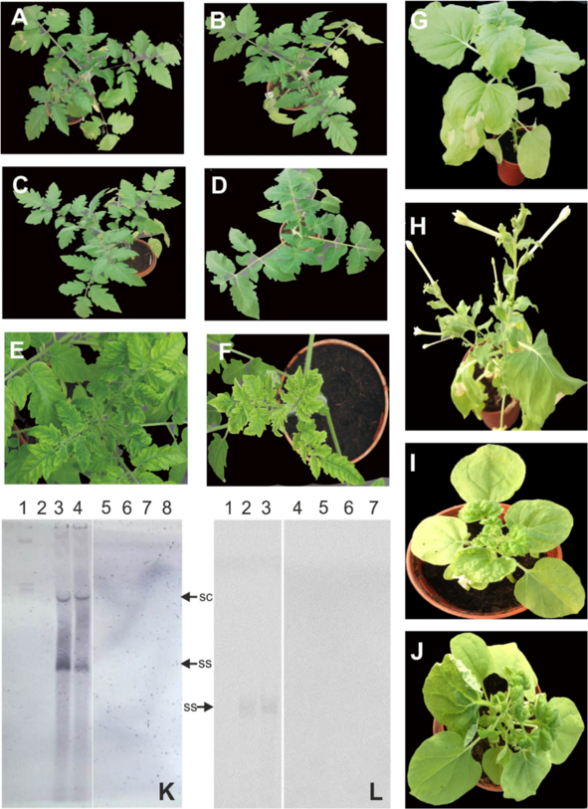
**Resistance to infection by TYLCV-OM and TYLCV-OM/ToLCB-OM imparted by the transient expression of the hpRNAi construct.** Tomato plants inoculated with TYLCV-OM and the hpRNAi construct **(A and B)**, TYLCV-OM/ToLCB-OM and the hpRNAi construct **(C and D)**, TYLCV-OM **(E)** or TYLCV-OM/ToLCB-OM **(F)**. *N. benthamiana* plants inoculated with TYLCV-OM and the hpRNAi construct **(G)**, TYLCV-OM/ToLCB-OM and the hpRNAi construct **(H)**, TYLCV-OM/ToLCB-OM **(I)** or TYLCV-OM **(J)**. Photographs of plants were taken at 30 dpi. Southern blot probed for the presence of TYLCV-OM sequences **(K)**. The DNA samples run on the gel were extracted from a healthy, non-inoculated *N. benthamiana* plant (lane 2), *N. benthamiana* plants inoculated with TYLCV-OM (lane 3), hpRNAi and TYLCV-OM (lanes 7 and 8) and tomato plants inoculated with TYLCV-OM (lane 4) or hpRNAi and TYLCV-OM (lanes 5 and 6). The sample in lane 1 consisted of 50 ng of TYLCV-OM plasmid.With the exception of lane 1, approx. equal amounts (10 μg) of total DNA extract was loaded in each case. Southern blot probed for the presence of ToLCBV-OM sequences **(L)**. The DNA samples run on the gel were extracted from a healthy, non-inoculated *N. benthamiana* plant (lane 1) and *N. benthamiana* plants inoculated with TYLCV-OM/ToLCB-OM (lane 2) or hpRNAi and TYLCV-OM/ToLCB-OM (lanes 6 and 7) and tomato plants inoculated with TYLCV-OM/ToLCB-OM (lane 3) or hpRNAi, TYLCV-OM/ToLCB-OM (lanes 4 and 5). Approx. equal amounts (10 μg) of total DNA extract were loaded in each case. The positions of the viral single-stranded (ss) and supercoiled (sc) replicative DNA forms are indicated.

*N. benthamiana* plants inoculated with TYLCV-OM or TYLCV-OM/ToLCB-OM showed the first symptoms of infection at15 days post inoculation (dpi) and all plants were ultimately symptomatic by 30 dpi (Table 1). The symptoms consisted of mild leaf curling which gradually increased in severity. By 30 dpi plants showed severe stunting, leaf curling, vein swelling and foliar yellowing (Figure 1, panels I and J). Overall the symptoms for TYLCV-OM/ToLCB-OM infected plants were more severe than those of TYLCV-OM inoculated plants, with leaves being smaller with more pronounced leaf curling, yellowing and vein swelling.

**Table 1:** **Effect of the transient expression of the hpRNAi construct on the infectivity of TYLCV-OM and TYLCV-OM/ToLCB-OM in** ***Nicotianabenthamiana*** **plants**

**Inoculum**	**Experiment**	**Infectivity (plants symptomatic/inoculated)**			SS*	**% Resistance**	**Molecular diagnosis**		
		**15 dpi**	**30dpi**	**40 dpi**			**PCR**	**Southern for TYLCV-OM**	**Southern for ToLCB-OM**
**Non-inoculated**	-	0/10	0/10	0/10	0	NA	_	_	_
**TYLCV-OM**	**I**	10/10	10/10	10/10	3	-	10/10	10/10	_
	**II**	10/10	10/10	10/10	3	-	10/10	10/10	_
	**III**	10/10	10/10	10/10	3	-	10/10	10/10	_
**TYLCV-OM/ ToLCB-OM**	**I**	10/10	10/10	10/10	3	-	10/10	10/10	10/10
	**II**	10/10	10/10	10/10	3	-	10/10	10/10	10/10
	**III**	10/10	10/10	10/10	3	-	10/10	10/10	10/10
**TYLCV-OM + hpRNAi**	**I**	0/10	1/10	2/10	1	80	6/10	0/10	0/10
	**II**	0/10	2/10	2/10	1	80	4/10	0/10	0/10
	**III**	0/10	3/10	4/10	2	60	6/10	1/10	0/10
**TYLCV-OM/ToLCB-OM + hpRNAi**	**I**	0/10	3/10	4/10	1	60	7/10	0/10	0/10
	**II**	0/10	3/10	3/10	2	70	6/10	3/10	2/10
	**III**	0/10	2/10	3/10	2	70	8/10	4/10	4/10

*Symptom severity for symptomatic plants rated according to AVDRC disease severity scale (0: Normal healthy plant, 1: light leaf yellowing, 2: moderate plant stunting with leaf yellowing and curling, 3: Severe plant stunting with leaf curling and yellowing).

In contrast the majority of *N. benthamiana* plants co-inoculated with the hpRNAi construct and either TYLCV-OM or TYLCV-OM/ToLCB-OM remained symptomless (Table 1). Overall more plants were symptomatic when the virus was inoculated with the betasatellite (10/30) than when the virus was inoculated alone (8/30). Analysis of inoculated *N. benthamiana* plants by Southern blot hybridization showed hybridization of both the virus and the betasatellite probes to DNA samples extracted from plants inoculated with the hpRNAi construct and either TYLCV-OM or TYLCV-OM/ToLCB-OM (Table 1) for a small number of plants; 1 out of 30 and 7 out of 30, respectively. Again, more plants were positive from inoculation with the virus and betasatellite than with just the virus. PCR diagnostics indicated that for plants co-inoculated with the hpRNAi construct, the majority of the plants contained virus but that the presence of the betasatellite increased the number of plants that ultimately were virus positive; 16 out of 30 for TYLCV-OM inoculated plants and 21 out of 30 for TYLCV-OM/ToLCB-OM inoculated plants. A Southern blot analysis of the PCR positive, non-symptomatic, hpRNAi construct and either TYLCV-OM or TYLCV-OM/ToLCB-OM inoculated plants is shown in Figure 1, panels K and L. This shows no hybridization, suggesting that virus and betasatellite levels in these plants were below the threshold for detection by hybridization.

All tomato plants co-inoculated with the hpRNAi construct and TYLCV-OM, in the absence or presence of ToLCB-OM, remained symptomless (Table 2; Figure 1, panels A-D). These plants continued to grow normally and flower. In contrast, non-transgenic tomato plants developed foliar yellowing and mild leaf curling by 30 dpi when inoculated with TYLCV-OM (Figure 1, panel E), while plants inoculated with TYLCV-OM/ToLCB-OM developed more severe foliar yellowing, stunting and leaf curling by 30 dpi (Figure 1, panel F).

**Table 2:** **Effect of the transient expression of the hpRNAi construct on the infectivity of TYLCV-OM and TYLCV-OM/ToLCB-OM in tomato cv Pusa Ruby plants**

**Treatments**	**Experiment**	**Infectivity (plants symptomatic/inoculated)**			SS*	**% Resistance**	**Molecular diagnosis**		
		**15 dpi**	**30 dpi**	**40 dpi**			**PCR**	**Southern for TYLCV-OM**	**Southern for ToLCB**
**Non-inoculated**		0/10	0/10	0/10	0	NA	_	_	_
**TYLCV-OM**	**I**	0/10	10/10	10/10	3	-	10/10	10/10	_
	**II**	0/10	10/10	10/10	3	-	10/10	9/10	_
	**III**	0/10	10/10	10/10	3	-	10/10	10/10	_
**TYLCV-OM/ToLCB-OM**	**I**	0/10	10/10	10/10	3	-	10/10	10/10	8/10
	**II**	0/10	10/10	10/10	3	-	10/10	9/10	8/10
	**III**	0/10	10/10	10/10	3	-	10/10	10/10	9/10
**TYLCV-OM + hpRNAi**	**I**	0/10	0/10	0/10	0	100	0/10	0/10	0/10
	**II**	0/10	0/10	0/10	0	100	0/10	0/10	0/10
	**III**	0/10	0/10	0/10	0	100	0/10	0/10	0/10
**TYLCV-OM/ToLCB-OM + hpRNAi**	**I**	0/10	0/10	0/10	0	100	0/10	0/10	0/10
	**II**	0/10	0/10	1/10	1	90	1/10	0/10	0/10
	**III**	0/10	0/10	0/10	0	100	0/10	0/10	0/10

*Symptom severity for symptomatic plants rated according to AVDRC disease severity scale (0: Normal healthy plant, 1: light leaf yellowing, 2: moderate plant stunting with leaf yellowing and curling, 3: severe plant stunting with leaf curling and yellowing).

Southern blot analysis of tomato plants detected viral DNA forms typical of geminivirus replication for plants inoculated with TYLCV-OM and TYLCV-OM/ToLCB-OM (Figure 1, panel K). Blots probed for the presence of the betasatellite showed hybridization of ssDNAfor TYLCV-OM/ToLCB-OM inoculated tomato (Figure 1, panel L). In contrast, for none of the tomato plants inoculated with either TYLCV-OM or TYLCV-OM/ToLCB-OM in the presence of the hpRNAi construct was hybridization to either virus or betasatellite probes detected. With the exception of a single plant, TYLCV-OM and ToLCB-OM were not detected by PCR in tomato plants co-inoculated with the hpRNAi construct, whereas specific DNA bands indicative of the virus/betasatellite were produced for PCR reactions containing DNA extracted from symptomatic plants that had been inoculated with TYLCV-OM or TYLCV-OM/ToLCB-OM but without the hpRNAi construct (Table 2).

### Production of transgenic *S. lycopersicum* cv. Pusa Ruby plants

*S. lycopersicum* L.cv. Pusa Ruby plants were transformed with the hpRNAi construct by *Agrobacterium-*mediated transformation. A total of 11 kanamycin resistant, primary transformed tomato plants were obtained. However, PCR analysis using primers CS-For/CS-Rev (Table 3), directing the amplification of an approx. 1100 bp fragment of the chalcone synthase intron, indicated that only 9 plants contained the transgene. These 9 plants were progressed to the T_1_ generation by self-pollination and used for evaluation of resistance against TYLCV-OM and TYLCV-OM/ToLCB-OM. All putative transgenic lines (20 seed of each line) were germinated on 500 mg/L kanamycin selection medium before transfer to soil in pots.

**Table 3:** **Oligonucleotide primers used in the study**

**Primer**	Sequence*
CS-For	5′-CCGACGAATTGTGGGAAGGT-3′
CS-Rev	5′-GCATAGCATGCAAAAACCCTCA-3′
FD-CP-382	5′-CTSARCTTCGACAGCCCXTA-3′
RD-CP-1038	5′-TGMGTACAXGCCATATACAA-3′
Sat01	5′-GGTACCACTACGCTACGCAGCAGCCGGT-3′
Sat02	5′-ACCTACCCTCCCAGGGGTACAC-3′
QF-OM	5′-GAAGCCCTGATGTTCCCCGTGG-3′
QR-OM	5′-CGATTTAACACAGAACCTCTTACC-3′

*The ambiguity codes used are X = A or T or G or C; S = G or C; R = A or G; M = C or A.

### Transgenic tomato plants harboring the hpRNAi construct are resistant to TYLCV-OM and the TYLCV-OM/ToLCB-OM complex

**Figure 2:**
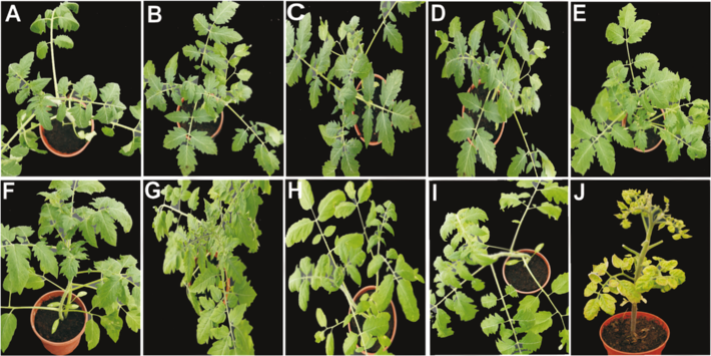
**Analysis of the resistance imparted by the hpRNAi construct in transgenic tomato to infection by TYLCV-OM.** Plants of transgenic tomato cv. Pusa Ruby lines 11, 12, 13, 18, 21, 23, 41, 51 and 52 inoculated with TYLCV-OM **(A to I)**. A TYLCV-OM inoculated wild-type Pusa Ruby plant is shown for comparison **(J)**. Photographs were taken at 60 dpi.

**Figure 3:**
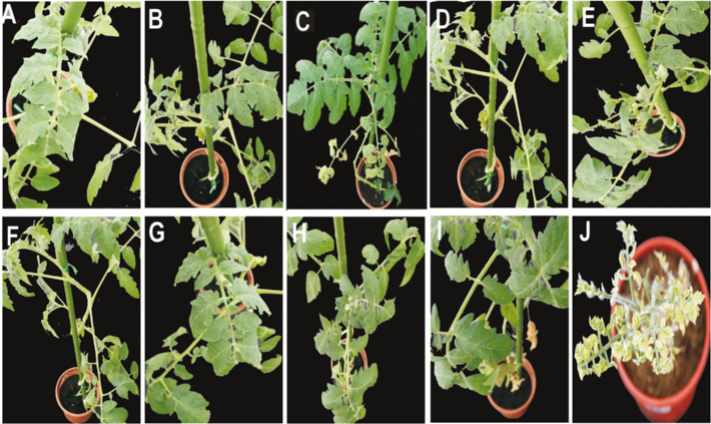
**Analysis of the resistance imparted by the hpRNAi construct in transgenic tomato to infection by TYLCV-OM/ToLCB-OM.** Plants of transgenic tomato cv. Pusa Ruby lines 11, 12, 13, 18, 21, 23, 41, 51 and 52 inoculated with TYLCV-OM/ToLCB-OM **(A to I)**. A TYLCV-OM/ToLCB-OM inoculated wild-type Pusa Ruby plant is shown for comparison **(J)**. Photographs were taken at 60 dpi.

All non-transgenic wild type *S. lycopersicum* cv. Pusa Ruby plants inoculated with TYLCV-OM (Figure 2 panel J) and TYLCV-OM/ToLCB-OM (Figure 3 panel J) developed severe yellowing, leaf curling and a reduced leaflet size, symptoms typical of this virus, by 30 dpi (Table 4). Such plants ceased to grow, failing to flower and produce fruit. Plants inoculated with TYLCV-OM/ToLCB-OM exhibited more severe symptoms than plants inoculated with only the virus, with significantly smaller leaflets.

**Table 4:** **Evaluation of the response of transgenic tomato lines harbouring the hpRNAi construct to challenge with TYLCV-OM and TYLCV-OM/ToLCB-OM**

**Transgenic line**	**TYLCV-OM (30 dpi)**			**TYLCV-OM/ToLCB (30 dpi)**			**TYLCV-OM (60 dpi)**			**TYLCV-OM/ToLCB (60 dpi)**		
	**Plants symptomatic/inoculated**	SS*	**% Resistance**	**Plants symptomatic/inoculated**	SS*	**% Resistance**	**Plants symptomatic/inoculated**	SS*	**% Resistance**	**Plants symptomatic/inoculated**	SS*	**% Resistance**
**11**	2/20	1	90	4/20	1	80	5/20	1	75	6/20	1	70
**12**	2/20	1	90	4/20	1	80	4/10	2	80	7/20	2	65
**13**	1/20	1	95	6/20	1	70	1/20	1	95	7/20	1	65
**18**	2/20	1	90	4/20	2	80	5/20	2	75	7/20	2	65
**21**	0/20	0	100	5/20	1	75	4/20	1	80	7/20	1	65
**23**	1/20	1	95	2/20	1	90	5/20	1	75	9/20	1	55
**41**	0/20	0	100	4/20	1	80	0/20	0	100	4/20	1	80
**51**	2/20	1	90	4/20	2	80	4/20	1	80	7/20	1	65
**52**	0/20	0	100	2/20	1	90	4/20	1	80	5/20	1	75
NC**	0/10	-	-	0/10	-	-	0/10	-	-	0/10	-	-
PC***	10/10	1	0	10/10	2	0	10/10	3	0	10/10	3	0

The data presented in each case is the sum of two independent experiments with 10 plants each.

*Symptom severity rated according to AVDRC disease severity scale (0: Normal healthy plant, 1: light leaf yellowing, 2: moderate plant stunting with leaf yellowing and curling, 3: Severe plant stunting with leaf curling and yellowing).

**Non-transgenic tomato cv. Pusa Ruby plants inoculated with pGreen0029 vector with no insert.

***Non-transgenic tomato cv. Pusa Ruby plants inoculated with TYLCV-OM or TYLCV-OM/ToLCB.

The majority of inoculated transgenic plants remained symptomless. With the exception of plants of line 41, for which no plants inoculated with TYLCV-OM showed symptoms, all lines showed up to 25% of plants mildly symptomatic following inoculation with TYLCV-OM and up to 45% of plants for inoculation with TYLCV-OM/ToLCB-OM by 60dpi (Table 4). The symptoms exhibited were some mild leaf curling and mild yellowing for TYLCV-OM inoculated and mild leaf curling and mild yellowing with occasionally severe symptoms (severe yellowing and reduced leaflet size) on single branches (Figure 3, panels C to E) for TYLCV-OM/ToLCB-OM inoculated plants.

**Figure 4:**
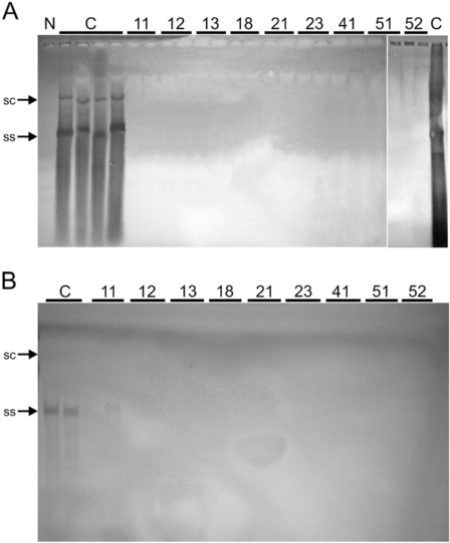
**Southern blot analysis of transgenic tomato plants harboring the hpRNAi construct inoculated with TYLCV-OM probed for the presence of TYLCV-OM (A) and inoculated with TYLCV-OM/ToLCB-OM probed for the presence of ToLCB-OM (B).** The DNA samples run on the gel were extracted from a non-transgenic non-inoculated tomato cv. Pusa Ruby plant (N), symptomatic TYLCV-OM (panel **A**) or TYLCV-OM/ToLCB-OM (panel **B**) inoculated non-transgenic Pusa Ruby plants (C) and two plants each of 9 transgenic Pusa Ruby lines (as indicated on the figure) inoculated with either TYLCV-OM (panel **A**) or TYLCV-OM/ToLCB-OM (panel **B**). Approx. 10 μg of total DNA was loaded on each case and the samples were extracted at 60 dpi. The positions of the viral single-stranded (ss) and supercoiled (sc) replicative DNA forms are indicated.

A Southern blot hybridization analysis of inoculated tomato plants detected high levels of both virus and betasatellite in symptomatic non-transgenic plants. However, with the exception of one plant, in which low levels of the betasatellite were detected, no virus or betasatellite DNA could be detected in inoculated transgenic plants (Figure 4).

#### Quantitative PCR determination of virus titer in plants

**Figure 5:**
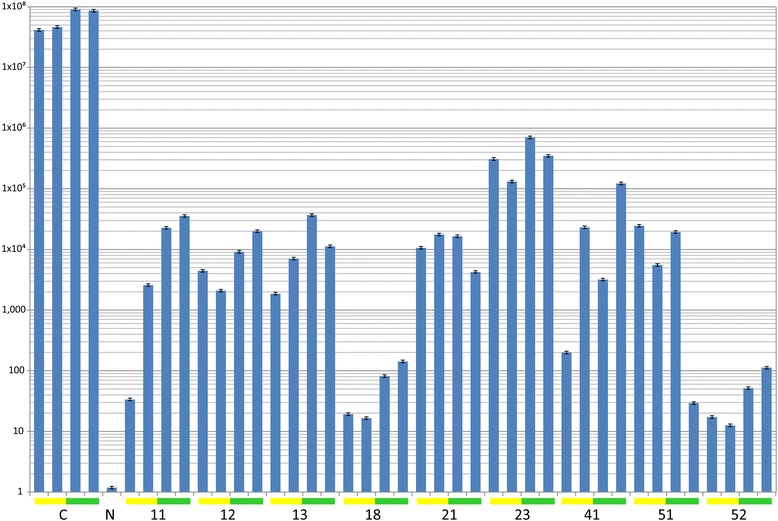
**Real-time quantitative PCR assay of TYLCV-OM in transgenic and non-transgenic tomato cv. Pusa Ruby plants inoculated with TYLCV-OM and TYLCV-OM/ToLCB-OM.** The bars indicate calculated virus titre (genome copies per 25 ng total DNA) in each case. The results are from qPCR reactions with DNA extracted from inoculated non-transgenic tomato cv. Pusa Ruby plants (C), a non-inoculated non-transgenic plant (N) and four plants each of 9 transgenic Pusa Ruby lines (as indicated on the figure) that were inoculated. For all the inoculated plants the left two bars (indicated by a yellow block at the base) are plants inoculated with only TYLCV-OM and the right two bars (indicated by a green block at the base) are plants inoculated with TYLCV-OM and ToLCB-OM. Each bar is the mean of three replicates of the qPCR reaction and the error bars indicate standard deviation. The leaf samples from which DNA was extracted were collected at 60 dpi.

The levels of virus in tomato plants inoculated with either TYLCV-OM or TYLCV-OM/ToLCB-OM were determined by a real time quantitative PCR (qPCR) assay on DNA samples extracted from plants at 60 dpi. The efficiency of qPCR reaction was 99.3% and a melt-curve analysis resulted in a single peak, indicative of the amplification of a single product. PCR reactions with DNA extracted from wild type non-inoculated tomato plants did not reach the threshold cycle (C_t_), indicative of no viral DNA in these samples. The qPCR analyses showed the presence of the virus in all inoculated tomato plants, including the transgenic plants. However, the level of virus was significantly lower in all transgenic plants than in the non-transgenic control plants (Figure 5). Overall the worst performing line (based on the qPCR results) was line 23 (with an <340 fold lower virus titre than non-transgenic plants) and the best performing lines were 18 and 52 (with a > 290,000 fold reduction). Also, for the majority of plants (both transgenic and non-transgenic) the levels of virus were significantly higher in plants inoculated with TYLCV-OM/ToLCB-OM than in plants inoculated with just the virus.

## Discussion

The results presented here have shown that the hpRNA approach to generating resistance in plants, targeted against four parts of the virus genome, has the potential to yield resistance against the begomovirus TYLCV-OM; a virus causing significant losses to tomato production in Oman. The majority of inoculated transgenic plants remained symptomless with one line, line 41, showing 100% resistance (no plants with symptoms) after inoculation with TYLCV-OM and 80% resistance following inoculation with TYLCV-OM/ToLCB-OM. However, the qPCR analysis showed all inoculated plants to contain virus but at levels significantly below the titer of non-transgenic plants. The level of resistance obtained here is thus best described as a “tolerance” to infection. This finding is consistent with a number of studies that have investigated transgenic resistance to geminiviruses using the RNAi approach [36-38].

For the majority of transgenic plants as well as the non-transgenic plants inoculated in the presence of the betasatellite the virus titer was higher than in plants inoculated with the virus alone. This is consistent with what is known about betasatellites which, in most cases, increase helper virus DNA levels in plants [39,40]. The precise reason for the increase in virus DNA levels induced by betasatellites is unclear. This could be due to the activity of the only protein expressed by betasatellites, known as βC1, which is a suppressor of gene silencing and is also believed to play a part in virus movement in plants [41,42].

Mubin et al. [43] have shown that betasatellites have the potential to overcome RNAi mediated resistance against begomoviruses. This work showed that an hpRNAi construct containing sequences of the Rep, TrAP and REn of the monopartite begomovirus *Cotton leaf curl Multan virus* could prevent symptomatic infection by transient assay in *N. benthamiana*, but that in the presence of the cognate betasatellite, Cotton leaf curl Mutan betasatellite, a significant number of plants developed symptoms. The results presented here are consistent with this. For all the transgenic lines, the presence of the betasatellite resulted in more symptomatic plants with more severe symptoms. This is likely due to the betasatellite increasing virus titre above the threhold level for inducing symptoms and the fact that betasatellites encode a dominant symptom (pathogenicity) determinant [44].

It remains unclear whether RNAi will be able to deliver effective resistance, which is durable in the field, against geminiviruses. The transgenic beans in Brazil with engineered resistance to BGMV are so far the only RNAi-based resistance to a plant infecting DNA virus that has been commercialized. This resistance is reported to be at immunity level [34,35,45]. For all other reported efforts to produce RNAi-based resistance in plants to begomoviruses the results have been less successful, usually resulting in tolerant plants that contain low levels of viral DNA and are non-, or very mildly, symptomatic [36-38]. RNA silencing in plants involves RNA-directed DNA methylation (RdDM), in which DNA homologous to a triggering RNA is methylated *de novo* leading to TGS [32,46]. This is believed to be, amongst other things, an adaptive defense mechanism against nuclear DNA viruses [29,47]. As a counter-defense geminiviruses have evolved proteins capable of suppressing methylation, which may explain why the development of RNAi-based resistance against geminiviruses has proven so problematic. The begomovirus-encoded suppressors of TGS include Rep, TrAP, V2 and the betasatellite-encoded βC1 [9,48-50]. The RNAi construct produced here was designed to both induce TGS (by including promoter sequences present in the IR) and post-transcriptionally silence two of the genes of proteins known to suppress TGS (V2 and Rep). Further studies will be required to determine whether TGS is occurring in the transgenic tomato lines.

A drawback of RNAi-mediated resistance is that it is sequence homology based. Thus, the resistance will only be effective against viruses with high levels of sequence identity across the targeted region [51]. In Oman at least five distinct begomoviruses cause disease in tomato [18,52-55]. Given that the ultimate goal is to produce tomato lines with a broad-spectrum resistance to all tomato infecting viruses occurring in Oman, the potential of the RNAi construct produced here to counter infection by the heterologous viruses should be investigated. However, it would seem likely that either separate constructs for each virus or a single construct, but utilizing sequences conserved between all the viruses, would be required. Investigation of these problems will be the objective of future studies.

## Materials and methods

### Production of the hpRNAi construct

A modified pGreen 0029 binary vector [56] was produced by designing a multiple cloning site (MCS) containing restriction and homing endonuclease recognition sequences (Additional file 1: Figure S1). The 237 bp MCS was synthesized by GenScript Inc. (New Jersey, USA) and provided in the plasmid vector pUC57. The modified MCS was excised on flanking *Eco*RV sites and cloned into pGreen 0029.

The hpRNAi construct consisted of 574 bp of sequence derived from TYLCV-OM (acc. no. DQ644565) cloned sense (virion-sense, with respect to the virus) and antisense (complementary-sense) separated by a chalcone synthase intron (sequence derived from the binary vector pFGC5941; acc. no. AY310901), with upstream a double CaMV 35S promoter and downstream a CaMV 35S terminator sequence (both derived from the binary vector pEAQ-HT [acc. no. GQ497234]). The 574 bp TYLCV-OM derived sequence consisted of a 175 bp fragment of the Rep gene (TYLCV-OM coordinates 1760-1934; antisense orientation with respect to the Rep gene) and a 399 bp fragment spanning the 3′ part of the IR, the 5′ ends of the V2 and overlapping CP genes (TYLCV-OM coordinates131-418).

The complete construct (4097 bp) consisting of 2x35S promoter, sense TYLCV sequence, chalcone synthase intron, antisense TYLCV sequence and 35S terminator, flanked by I-*Ceu*I and PI-*Psp*I/PI-*Sce*I sites, was synthesized by GenScript and supplied in pUC57 (Additional file 2: Figure S2). The expression cassette (4067 bp) was then excised using I-*Ceu*I and PI-*Psp*I and ligated into the modified pGreen0029 binary vector.

### Tomato transformation

The modified pGreen0029 vector containing the hpRNAi construct was transformed by electroporation in *Agrobacterium tumefaciens* AGL1. *Agrobacterium*-mediated transformation of tomato cv. Pusa Ruby was conducted as described earlier [57]. Nine primary transformant plants were confirmed to contain the hpRNAi construct by PCR with primer pair CS-For/CS-Rev which are designed to amplify the chalcone synthase intron fragment from the construct (Table 3). The 9 plants were self-pollinated and seed was collected. The seed were germinated on MS basal medium containing 500 mg/L kanamycin and unbleached seedlings were transferred to pots containing autoclaved soil and maintained in a glasshouse at 28-29°C and 80-90% relative humidity. These T_1_ generation transgenic plants were used for virus inoculation.

### *Agrobacterium*-mediated inoculation of plants with TYLCV-OM and ToLCB-OM

Agro-infectious clones TYLCV-OM (acc. no. DQ644565) and ToLCB-OM (acc. no.HE800544) were used to infect tomato plants. Three leaves per plants were inoculated. Non-transgenic Pusa Ruby tomato plants of the same age were infiltrated with TYLCV-OM and TYLCV-OM/ToLCB-OM as positive controls. All plants were maintained in an insect-free glasshouse and monitored daily for the appearance of symptoms. Leaf samples from all plants were collected at 60 dpi. Total genomic DNA was isolated from leaf tissues by the CTAB method [58]. The presence of TYLCV-OM and ToLCB-OM was assessed by PCR using primer pairs FD-CP-382/RD-CP-1038 and Sat01/Sat02, respectively (Table 3).

### Southern hybridization

DNA samples (~10 μg) were electrophoresed in 1% agarose gels in 1X TBE buffer at 60-80 volts for 5 to 6 hours. DNA was transferred to Hybond N+ (Amersham) nylon membrane by capillary transfer in 10 X SSC. The membrane was air dried, UV cross-linked and stored at 4°C until use between two sheets of wet Whatman filter paper.

For detection of the virus a 650 bp fragment of the CP gene of TYLCV-OM was PCR amplified by using primers FDCP/RDCP.A 1084 bp*Bam*HI- *Xba*I fragment of the ToLCB-OM clone was gel purified for the detection of the betasatellite. These DNA fragments were labeled with digoxigenin using a DIG-High Prime DNA Labeling and Detection Starter Kit I (Roche GmbH, Germany).

Blots were hybridized with the respective probes at 42°C for 12-16 hours. Unbound probe was removed by washing with 2XSSC, 0.1% SDS and 1X SSC, 0.1%SDS for 30 min each. Hybridization of probes was detected using CDP star (Roche) and X-ray film (AmershamHyperfilm, GE Life Sciences) according to the manufacturer’s instructions.

### Quantitative real time PCR

Primer pair QF-OM and QR-OM, which amplify a 50 bp product of the TYLCV-OM CP gene, was used for quantification of the virus (Table 3). The copy number of virus was calculated by reference to a standard curve obtained by serial dilution of a plasmid containing the full-length TYLCV-OM (acc. no. DQ644565). Reaction mixes consisted of 10 μl of 1XPower SYBR Green master mix (Life Technologies, USA), 0.10 μl of each primer (10 pmol) and 2.5 μl of DNA sample (10 ng/μl) in a total volume of 25 μl.

PCR reactions were carried out in clear optical plates inan Applied Biosystems 7500 (Life Technologies) real time PCR detection system. The machinewas programmed for 1 cycle at 94°C for 5 min, followed by 35 cycles of 30s at 94°C, 30s at 55°C and 30s at 72°C. All reactions were conducted in triplicate. Data analysis was performed using Applied Biosystems 7500 software version 2.0.6.

## Additional files

Additional file 1:
**Modified MCS in pGreen vector.**
Additional file 2:
**Multigene hpRNAi cassette for TYLCV-OM.**
