# Supplementary material for: RNA interference-based resistance in transgenic tomato plants against Tomato yellow leaf curl virus-Oman (TYLCV-OM) and its associated betasatellite
Source: Virol J. 2015 Mar 4;12:38. doi: 10.1186/s12985-015-0263-y (PMC4359554; doi:10.1186/s12985-015-0263-y)
Supplement: Additional file 1: — Modified MCS in pGreen vector. [file 12985_2015_263_MOESM1_ESM.doc]

MCS for modified pGreen:

**EcoRV/SgfI/SwaI/AscI/I-PpoI/I-SceI/I-CeuI/PI-PspI/PI-SceI/**

**PI-TliII/PacI/SrfI/EcoRV**

MCS Sequence (237 bp):

gatatcGCGATCGCATTTAAATGGCGCGCCTAACTATGACTCTCTTAAGGTAGCCAAATAGTTACGCTAGGGATAACAGGGTAATATAGCGTAACTATAACGGTCCTAAGGTAGCGAATGGCAAACAGCTATTATGGGTATTATGGGT**AAATTGCTTGCAAACAGCTATTACGGCTAT**ATCTATGTCGGGTGCGGAGAAAGAGGTAATGAAATGGTTAATTAAGCCCGGGCgatatc
