# Supplementary material for: RNA interference-based resistance in transgenic tomato plants against Tomato yellow leaf curl virus-Oman (TYLCV-OM) and its associated betasatellite
Source: Virol J. 2015 Mar 4;12:38. doi: 10.1186/s12985-015-0263-y (PMC4359554; doi:10.1186/s12985-015-0263-y)
Supplement: Additional file 2: — Multigene hpRNAi cassette for TYLCV-OM. [file 12985_2015_263_MOESM2_ESM.doc]

**Multigene hpRNAi cassette for TYLCV-OM**

**I-CeuI/2x35S/(virion-sense)Rep:IR-V2-CP/AvrII/chalcone synthase intron/SmaI/ScaI/(complementary-sense)CP-V2-IR:Rep/35S terminator/PI-PspI/PI-SceI(4097 bases)**

TAACTATAACGGTCCTAAGGTAGCGAAACATGCTGAAATCACCAGTCTCTCTCTACAAATCTATCTCTCTCTATTTTCTCCATAAATAATGTGTGAGTAGTTTCCCGATAAGGGAAATTAGGGTTCTTATAGGGTTTCGCTCATGTGTTGAGCATATAAGAAACCCTTAGTATGTATTTGTATTTGTAAAATACTTCTATCAATAAAATTTCTAATTCCTAAAACCAAAATCCAGTACTAAAATCCAGATCTCCTAAAGTCCCTATAGATCTTTGTCGTGAATATAAACCAGACACGAGACGACTAAACCTGGAGCCCAGACGCCGTTCGAAGCTAGAAGTACCGCTTAGGCAGGAGGCCGTTAGGGAAAAGATGCTAAGGCAGGGTTGGTTACGTTGACTCCCCCGTAGGTTTGGTTTAAATATGATGAAGTGGACGGAAGGAAGGAGGAAGACAAGGAAGGATAAGGTTGCAGGCCCTGTGCAAGGTAAGAAGATGGAAATTTGATAGAGGTACGCTACTATACTTATACTATACGCTAAGGGAATGCTTGTATTTATACCCTATACCCCCTAATAACCCCTTATCAATTTAAGAAATAATCCGCATAAGCCCCCGCTTAAAAATTGGTATCAGAGCCATGAATAGGTCTATGACCAAAACTCAAGAGGATAAAACCTCACCAAAATACGAAAGAGTTCTTAACTCTAAAGATAAAAGATCTTTCAAGATCAAAACTAGTTCCCTCACACCGGAATGCATTGCCAGTCCCTCTGGGCCCCCATTAATTCCTTGAAGTGCTTTAAATAATGCGGGTTTACGTCATCAATGACGTTAAACCACGCATCATTACTGTATACCTTTGGACTTAGGTCTAGATGTCCACATAAATAATTATGTGGGCCTAGAGACCTGGCCCACATTGTTTTGCCTGTTCCACGTGGGTCCCACACACGTCGCTATCATCCAATCAAATTGATGACTGAAACGTTAGATAATTGTTTATTTGTCTTTATAAACTTAGTCCCCAAGTTTGTTGTCTTGCAATATGTGGGATCCACTTCTTAATGAATTTCCTGAATCCGTTCACGGATTTCGTTGTATGTTAGCTATTAAATATTTGCAGGCCGTTGAGGAAACGTATGAGCCCAACACTTTGGGCCACGATTTAATTAGGGATCTTATATCTGTTGTAAGGGCTCGTGACTATGTCGAAGCGACCCGGCGATATAATCATTTCCACGCCCGTCTCGAAGGTTCGCCGAAGGCTGAACTTCGACAGCCCCTACAGCAGCCGTGCTGCTGCCCCCATTGTCCAAGGCATAAACAAGCGCCGCCTAGGGTGTAAGAATTTCTTATGTTACATTATTACATTCAACGTTTTATCTTAATTGGCTCTTCATTTGATTGAAATTTGACAATTATTTCTTGTTTTTTTTTTTGTCACACTCTTTTTGGGTTGGGGTGGCCGACGAATTGTGGGAAGGTAGAAAGAGGGGAGGACTTTTGTTATACTCCATTAGTAATTACTGTTTCCGTTTCAATTTATGTGACAATATTTCCTTTTTAGTCGGTTCCAAAAGAAAATGTCAGCATTATAAACAATTTAATTTTGAAATTACAATTTTGCCATTAATAAAATGATTTACAACCACAAAAGTATCTATGAGCCTGTTTGGGTGGGCTTATAAGCAGCTTATTTTAAGTGGCTTATAAGTCAAAAAGTGACATTTTTGAGAAGTTAGAAAATCCTAACTTCTCAAAAAGTAGCTTTTAAGCCACTTATGACTTATAAGTCCAAAAATTTTTAAGTTACCAAACATATATTAATGGGTTTATAAGCTTATAAGCCACTTTTAAGCTCACCCAAACGGGTTCTATGTCTCACTTTAGACTACAAATTTTAAAAGTCTTCATTTATTTCTTAATCTCCGTGGCGAGTAAACTATAACACATAAAGTGAAACGGAGGGAATAAGATGGAGTCATAAACTAATCCAAATCTATACTCTCTCCGTTAATTTGTTTTTTAGTTTGATTTGGTACATTAATAAAACAGATTTTTCGAAGGTTATAAACACAGACAGATGTTTCCCAGCGAGCTAGCAAAATTCCAAGATTTCTGTCGAAAATTCGTGTGTTTCTAGCTAGTACTTGATGTTATCTTTAACCTTTTAGTAATTTTTTGTCCTTTTCTTTCTATTTTTCATCTTACAATGAATTATGAGCAAGTTCCTTAAGTAGCATCACACGTGAGATGTTTTTTATGATATTGACTAAATCCAATCTTTACCATTCCTTAACTAGTAAAATACAACACATGTTAATTGATACATTGCTTAACACTGAGGTTAGAAAATTTTAGAAATTAGTTGTCCAAATGCTTTGAAATTAGAAATCTTTAATCCCTTATTTTTTTTTAAAATGTTTTTTCTCACTCCAAAGAAAGAGAAACTGACATGAAAGCTCAAAAGATCATGAATCTTACTAACTTTGTGGAACTAAATGTACATCAGAATGTTTCTGACATGTGAAAATGAAAGCTCTTAATTTTCTTCTTTTATTTATTGAGGGTTTTTGCATGCTATGCATTCAATTTGAGTACTTTAAAGCACCTATAAACACTTACTTACACTTGCCTTGGAGTTTATGTTTTAGTGTTTTCTTCACATCTTTTTTGGTCAATTTGCAGGTCCGACCGCCCGGGAGTACTGGCGCTTGTTTATGCCTTGGACAATGGGGGCAGCAGCACGGCTGCTGTAGGGGCTGTCGAAGTTCAGCCTTCGGCGAACCTTCGAGACGGGCGTGGAAATGATTATATCGCCGGGTCGCTTCGACATAGTCACGAGCCCTTACAACAGATATAAGATCCCTAATTAAATCGTGGCCCAAAGTGTTGGGCTCATACGTTTCCTCAACGGCCTGCAAATATTTAATAGCTAACATACAACGAAATCCGTGAACGGATTCAGGAAATTCATTAAGAAGTGGATCCCACATATTGCAAGACAACAAACTTGGGGACTAAGTTTATAAAGACAAATAAACAATTATCTAACGTTTCAGTCATCAATTTGATTGGATGATAGCGACGTGTGTGGGACCCACGTGGAACAGGCAAAACAATGTGGGCCAGGTCTCTAGGCCCACATAATTATTTATGTGGACATCTAGACCTAAGTCCAAAGGTATACAGTAATGATGCGTGGTTTAACGTCATTGATGACGTAAACCCGCATTATTTAAAGCACTTCAAGGAATTAATGGGGGCCCAGAGGGACTGGCATCTAGACCGTCACCGGTGTGAGGGAACTAGTTTTGATCTTGAAAGATCTTTTATCTTTAGAGTTAAGAACTCTTTCGTATTTTGGTGAGGTTTTATCCTCTTGAGTTTTGGTCATAGACCTATTCATGGCTCTGATACCAATTTTTAAGCGGGGGCTTATGCGGATTATTTCTTAAATTGATAAGGGGTTATTAGGGGGTATAGGGTATAAATACAAGCATTCCCTTAGCGTATAGTATAAGTATAGTAGCGTACCTCTATCAAATTTCCATCTTCTTACCTTGCACAGGGCCTGCAACCTTATCCTTCCTTGTCTTCCTCCTTCCTTCCGTCCACTTCATCATATTTAAACCAAACCTACGGGGGAGTCAACGTAACCAACCCTGCCTTAGCATCTTTTCCCTAACGGCCTCCTGCCTAAGCGGTACTTCTAGCTTCGAACGGCGTCTGGGCTCCAGGTTTAGTCGTCTCGTGTCTGGTTTATATTCACGACAAAGATCTATAGGGACTTTAGGAGATCTGGATTTTAGTACTGGATTTTGGTTTTAGGAATTAGAAATTTTATTGATAGAAGTATTTTACAAATACAAATACATACTAAGGGTTTCTTATATGCTCAACACATGAGCGAAACCCTATAAGAACCCTAATTTCCCTTATCGGGAAACTACTCACACATTATTTATGGAGAAAATAGAGAGAGATAGATTTGTAGAGAGAGACTGGTGATTTCAGCGTACCGTCCGAACCCCTGGCAAACAGCTATTATGGGTATTATGGGTAAATTGCTTGCAAACAGCTATTACGGCTAT
